# Thoracoscopic enucleation of a large esophageal leiomyoma using a three thoracic ports technique

**DOI:** 10.1186/1477-7819-4-70

**Published:** 2006-10-04

**Authors:** Thawatchai Akaraviputh, Vitoon Chinswangwatanakul, Jirawat Swangsri, Varut Lohsiriwat

**Affiliations:** 1 Division of General Surgery, The Department of Surgery, Faculty of Medicine, Siriraj Hospital, Mahidol University, Bangkok 10700, Thailand

## Abstract

**Background:**

Video assisted thoracoscopic resection of an esophageal leiomyoma offers distinct advantages over an open approach. Many papers have described various techniques of thoracoscopic resection.

**Case presentation:**

We describe a 32-year old man who presented with intermittent dysphagia. Imaging studies showed a large esophageal leiomyoma. He underwent thoracoscopic enucleation using a three thoracic-ports technique.

**Conclusion:**

Thoracoscopic enucleation can be technically performed using a three thoracic-ports technique.

## Background

Leiomyoma is the most common benign esophageal tumor (70%–80%), occurring more often in the lower than the upper part. Many authors have described the use of videothoracoscopic surgery to performed enucleation of an esophageal leiomyoma with various techniques [1-3]. Everitt *et al *[4] was the first to report the thoracoscopic approach using seven thoracic ports. More recently, others have reported using the thoracoscopic approach with four thoracoscopic trocars or a small thoracotomy (Table 1). Herein, we report a patient with large esophageal leiomyoma, which was removed thoracoscopically, using a three thoracoscopic trocars technique.

**Table 1:** The list of publications reporting thoracosocpic enucleation technique for esophageal leiomyoma.

**Author**	**Year**	**Thoracoscopic technique**
Everitt *et al *[4]	1992	Right-sided approach: 7 trocars
Izumi Y *et al *[10]	1995	Right-sided approach: 6 trocars
Schmid *et al *[11]	1997	Right-sided approach: 4 trocars
Roviaro *et al *[1]	1998	Rtght-sided approach: 3 trocars with small thoracotomy
Infante *et al *[12]	2001	Left-sided approach: 4 trocars
Coral *et al *[9]	2003	Right-sided approach: 4 trocars
Rahden *et al *[13]	2004	Right/Left sided approach: 4 trocars
Our study	2006	Right-sided approach: 3 trocars

## Case presentation

**Figure 1:**
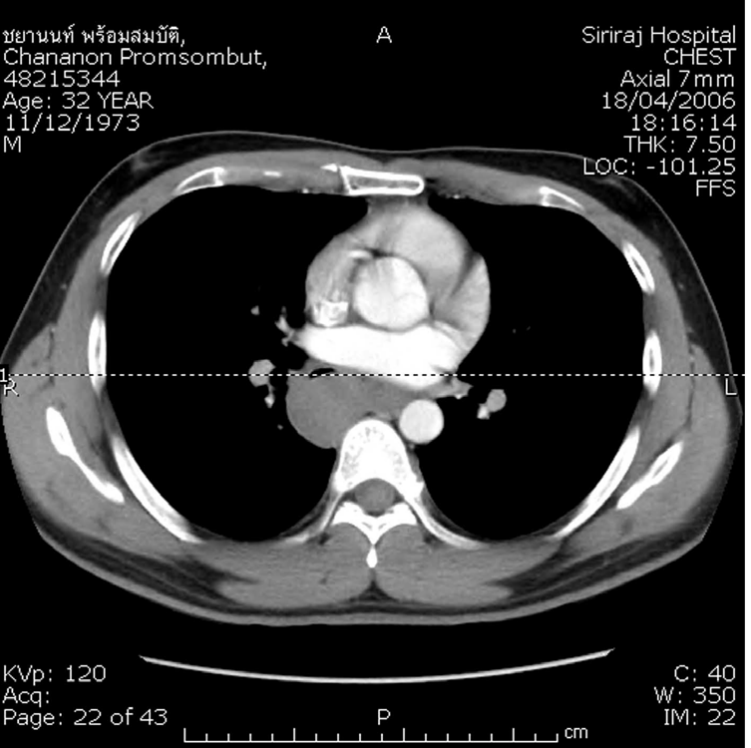
Computed tomography scan of the chest showing the esophageal tumor mass bulging toward the right pleural cavity.

A 32-year-old man presented with a 6-month history of intermittent dysphagia. Barium swallow showed an extrinsic compression of the lower thoracic esophagus. An upper gastrointestinal endoscopy was performed, which revealed a large submucosal mass in the lower thoracic esophagus without mucosal irregularity. Endoscopic ultrasonography (EUS) and computed tomography (CT) of the thorax showed a large homogeneous tumor in the esophageal muscular layer, measuring about 5 × 6 cm, without paraesophageal lymph nodes and projecting into the right pleural cavity (Figure 1). Routine laboratory and clinical findings were normal.

**Figure 2:**
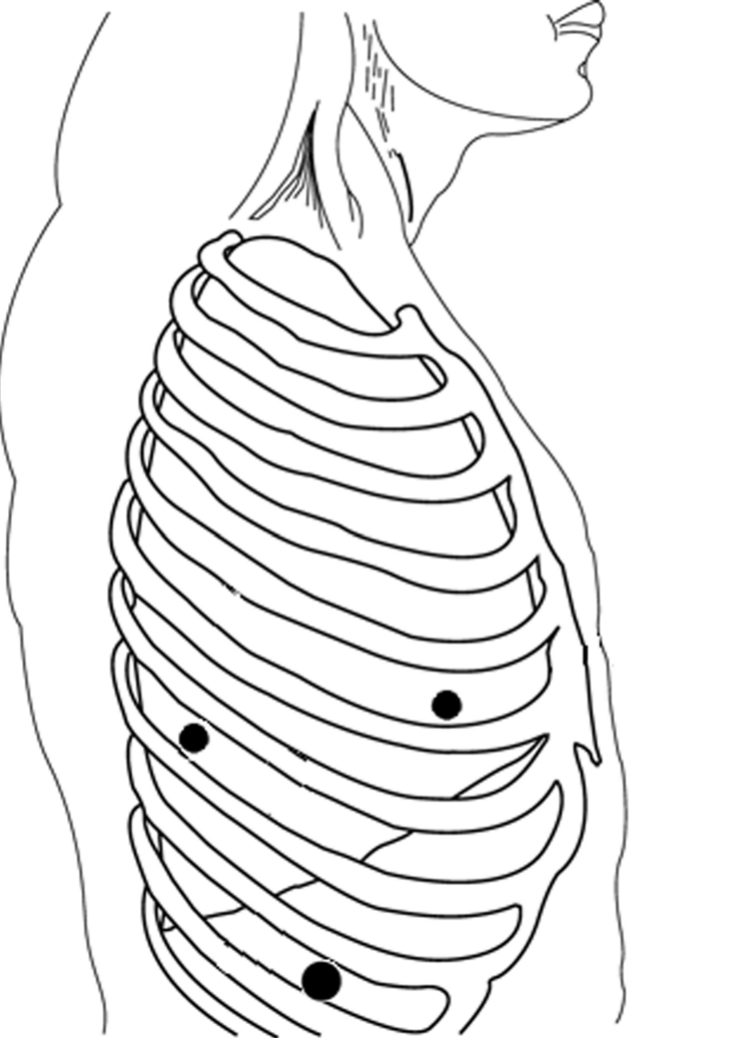
Patient positioning and port sites (A, B and C) for the right side. A: 5-mm port, posterior axillary line, seventh intercostal space. B: 5-mm port, anterior axillary line, fifth intercostal space. C: Camera port, mid-axillary line, nineth intercostal space.

The patient was intubated with a double lumen tube for one-lung ventilation and was positioned in the left lateral decubitus position. Three thoracic trocars were introduced (Figure 2). The camera port (10-mm) was placed at the ninth intercostal space, mid-axillary line. Two 5-mm trocars were introduced at the fifth intercostal space, anterior axillary line and the seventh intercostal space, posterior axillary line for using a "grasper" or coagulating device. The left lung was retracted to expose the lower thoracic esophagus. A simple hook was used to divide the mediastinal pleura overlying the esophagus. The lesion was then enucleated by careful dissection.

**Figure 3:**
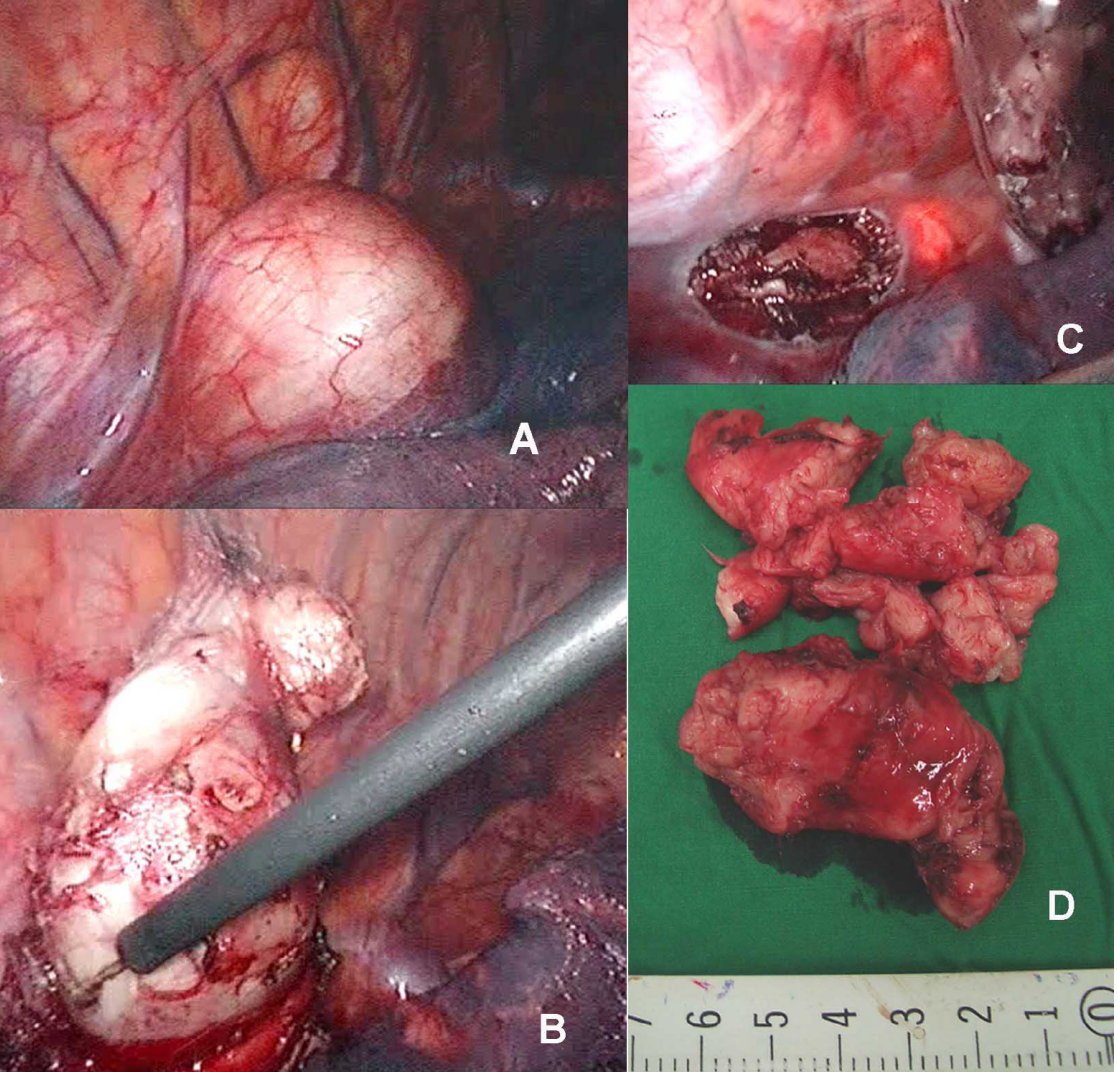
Thoracoscopic findings: (A) The esophageal tumor projects into the right thoracic space; (B) The tumor is enucleated with a simple hook-electrocautery; (C) Trans-illumination from intraopeative esophagoscopy was identified after the tumor was collected in a plastic bag; (D) The tumor was completely removed through camera port in small pieces.

Intraoperative endoscopy with air insufflation was performed to confirm esophageal integrity (Figure 3). The esophageal muscle was re-approximated with interrupted 3/0 Dexon. The specimen was removed within an endobag through the camera port. A 28 Fr chest tube was inserted through the camera port for postoperative drainage. The operative time was 2 hours and intraoperative blood loss was minimal.

Barium swallow at day 3 after surgery revealed no leakage and the patient was started on a liquid diet on day 4. The pathological report showed a leiomyoma with mitotic figure 0–1 per10 high power fields (HPF). Immunoperoxidase stainings were positive for smooth mucle actin, and negative for S-100, CD34 and C-kit. The patient was discharged on postoperative day 6. The patient is currently asymptomatic three months after surgery.

## Discussion

Esophageal leiomyoma is an uncommon benign tumor of smooth muscle origin. The most common anatomical location is in the lower third of the esophagus [5]. Malignant degeneration is rare, but removal is often required on symptomatic grounds. The characteristics of the lesion are clearly seen using esophagoscopy and conventional imaging techniques (barium swallow, CT scan, EUS) without the need for preoperative endoscopic biopsy [2]. Thoracoscopic enucleation is less invasive than open surgery, avoiding scaring and the discomfort of thoracotomy.

A thoracoscopic approach offers potential advantages compared with traditional thoracotomy, including minimal aesthetic disability, less pain, and better postoperative respiratory function. The limited operative trauma should allow a reduced postoperative hospital stay and more rapid resumption of normal activity [13].

The leiomyoma enucleation was easily performed and the esophageal muscular layer was carefully closed because of the reported development of a pseudodiverticulum after the procedure [6-8]. Intraoperative endoscopy with air insufflation confirmed an intact mucosal layer without any degree of esophageal stricture after suturing.

Some authors state that intraoperative endoscopy is not necessary to detect any perforation, because they infused blue dye proximally to the tumor after distal compression to create a bulge in the mucosa [9]. However, intraoperative endoscopy can reveal an electrical injury to mucosa which cause delayed perforation.

The advantages of thoracoscopic removal of esophageal leiomyoma are confirmed in the limited series that have been published in recent years. Of particular significance is the relative simplicity of this kind of approach compared with traditional thoracotomy [1]. Even with previous reports using four thoracic ports, the technique is applicable with three ports without any special instruments. This technique can be performed without morbidity and mortality, as in the recent study described.

## Conclusion

Thoracoscopic enucleation is treatment of choice of esophageal leiomyoma. Even with previous reports using four thoracic ports, the technique is replicable with only three ports.

## Competing interests

The author(s) declare that they have no competing interests.

## Authors' contributions

**TA **is the primary surgeon in charge who prepared the manuscript, **VC **is the second surgeon in charge who helped in the preparation of manuscript and edited it for its scientific content. **TA, JS **are the surgical staff who helped in the preparation of the figures for this manuscript. **VL **edited the manuscript for its scientific content.

All authors read and approved the final manuscript.
